# Digital counting of force accumulation during mechanotransduction

**DOI:** 10.52601/bpr.2024.240905

**Published:** 2024-06-30

**Authors:** Xinyu Zhang, Bei Liu

**Affiliations:** 1 National Biomedical Imaging Center, Peking University, Beijing 100871, China

The precise measurement of cellular forces at the single-molecule level is crucial for unlocking the secrets of mechanotransduction, where cells convert mechanical stimuli into biochemical signals. Existing approaches based on elastic peptides and DNA hairpins have provided insights into the “rupture forces” by converting sustained tensions into fluorescence signals. Yet, capturing the dynamics of how tension forces accumulate remains a formidable challenge.

Dr. Taekjip Ha’s laboratory at Harvard Medical School developed a highly sensitive single-molecule technique named Overstretching Tension Sensor (OTS) to measure the loading rates of forces exerted on cell adhesion molecules, specifically integrins (Jo *et al.* 2024). OTSs are composed of various oligonucleotides, which dehybridize at different levels of force depending on the length and GC content. Leveraging this principle, the team mapped the sequences of oligonucleotides to the magnitude of force. By repeatedly introducing quencher strands during imaging, OTS could maintain high detection sensitivity over prolonged periods (Fig. 1). Utilizing serially connected OTSs, the team determined that integrin loading rates in cells range from 0.5 to 4 piconewtons per second, with a notable threefold increase in leukocytes compared to epithelial cells. The research underscores the importance of understanding the physiological loading rate — how quickly the force is applied — to accurately determine the mechanical strength of receptor-ligand bonds in their functional contexts.

**Figure 1:**
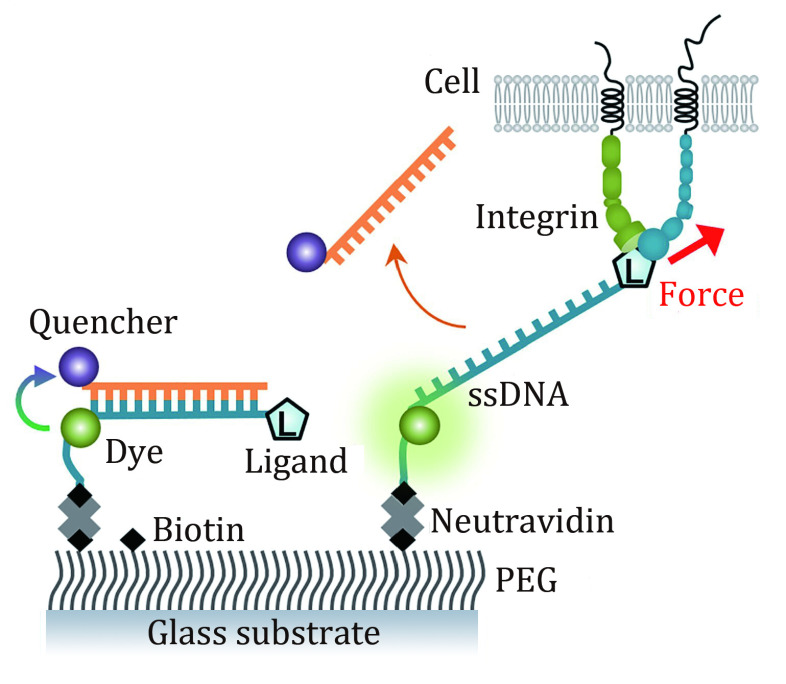
Schematic of force detection using OTS. The 5’ end of ssDNA is conjugated to cyclo-RGDfK to target αV integrins while its 3’ end is biotinylated and labeled with a Cy3 fluorophore. The quencher strand, conjugated with a fluorescent quencher (BHQ2), was introduced to form a DNA duplex to suppress the fluorescence signal. Once the quencher strand is removed by tension force, the fluorescence signal can be detected. ssDNA, single-stranded DNA. PEG, polyethylene glycol. Modified from Jo *et al*. (2024)

While this study has successfully addressed a crucial limitation, several significant challenges remain within the field: the reliance on 2D surfaces fails to capture the intricacies of the 3D environment; the urgent need for visualizing internal cellular forces and ultra-sensitive, accurate probes for *in vivo* force detection significantly impedes a thorough understanding of mechanotransduction.

## Conflict of interest

Xinyu Zhang and Bei Liu declare that they have no conflict of interest.
